# Offset fémoral et le fonctionnement de la hanche dans la prothèse totale de la hanche

**DOI:** 10.11604/pamj.2014.18.68.3186

**Published:** 2014-05-22

**Authors:** Abdelillah Rachid, Erraji Moncef, Abbassi Najib, Najib Abdeljaouad, Daudi Abdelkarim, Yacoubi Hicham

**Affiliations:** 1 Service de Traumatologie-Orthopédie, CHU Mohammed VI, Oujda, Maroc

**Keywords:** Prothèse, hanche, Offset fémoral, prosthesis, hip, femoral offset

## Abstract

L'offset fémoral est l’élément pronostic le plus important dans l'arthroplastie de la hanche. Sa restauration est devenue de plus en plus une obligation. Toute modification de la valeur native de l'offset retenti sur la qualité du fonctionnement clinique de la hanche. Le but de notre travail était d’évaluer le fonctionnement clinique de la hanche en fonction de la valeur de l'offset fémoral après une arthroplastie. C'est une étude rétrospective effectuée entre 2010 et 2013, comportant 27 patients ayant bénéficiés d'une arthroplastie totale de la hanche. Les patients ayant eu un geste chirurgicale antérieur sur la même hanche ou sur la hanche controlatérale ont été exclus. Les mesures ont été effectuées sur des radiographies standards de la hanche en rotation interne d'environ 15°, avec un agrandissement à 100%. Les résultats cliniques étaient évalués au moyen du score de WOMAC qui était en moyenne de 15,2 points, et le score de Merle d'Aubigné-Postel avec une moyenne de 15 points. Deux autres tests ont été évalués dans notre étude qui sont le step et le hop test. Les meilleurs résultats fonctionnels ont été obtenus chez les patients ayant eu une latéralisation de la tige fémoral avec un offset augmenté.

## Introduction

L'offset de la hanche est une variable définie qui traduit l’équilibre entre le poids du corps et la force de résistance fournie par les abducteurs de la hanche [1]. C'est la distance du segment perpendiculaire élevé du centre de rotation de la hanche à la ligne d'action des muscles abducteurs [2]. L'implantation des prothèses totales de la hanche chez des sujets jeunes nécessitent le respect d'un certains nombre de règles biomécaniques, afin d'assurer une pérennité des implants. Ainsi la restauration de l'offset fémoral permet d'améliorer l'amplitude des mouvements articulaires et surtout d'optimiser l'efficacité des muscles fessiers [3]. Notre travail a pour but d'analyser la relation entre la valeur de l'offset fémoral et le fonctionnement clinique de la hanche.

## Méthodes

C'est une étude rétrospective effectuée au service de traumatologie-orthopédie du CHU d'Oujda sur une période de 3,5 ans allant de Janvier 2010 à juin 2013. Elle comporte 27 patients ayant bénéficiés d'une arthroplastie totale de la hanche cimentées et non cimentées avec un couple de frottement métal-PE. Les patients ayant des antécédents de chirurgie sur l'une des deux hanches ont été exclus de cette étude. Ont été étudiés les paramètres épidémiologiques, cliniques, radiologiques avant et après l'implantation de la prothèse totale de la hanche. Les mesures de l'offset fémoral ont été effectuées sur des radiographies standards de la hanche en rotation interne d'environ 15°, avec un agrandissement à 100%.

## Résultats

**Figure 1:**
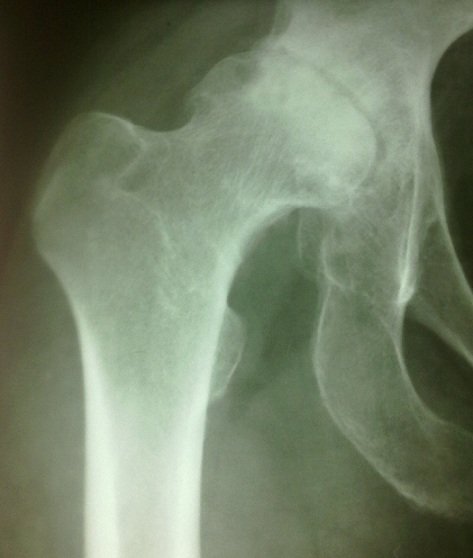
Image radiologique d'une coxarthrose primitive

**Figure 2:**
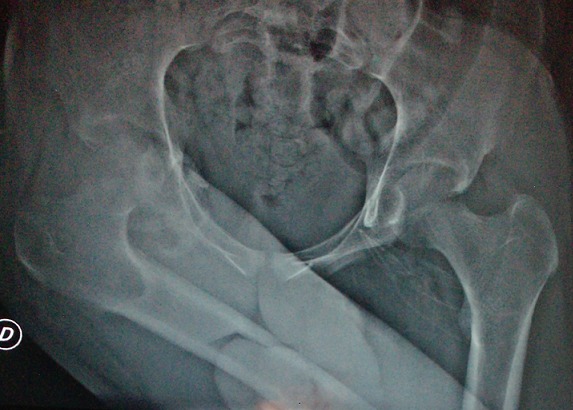
Image radiologique d'une luxation négligée de la hanche

**Figure 3:**
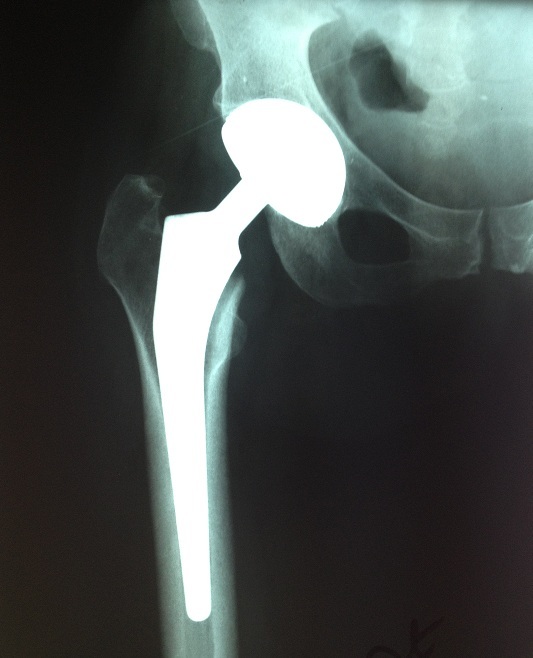
Contrôle radiologique post-opératoire

**Figure 4:**
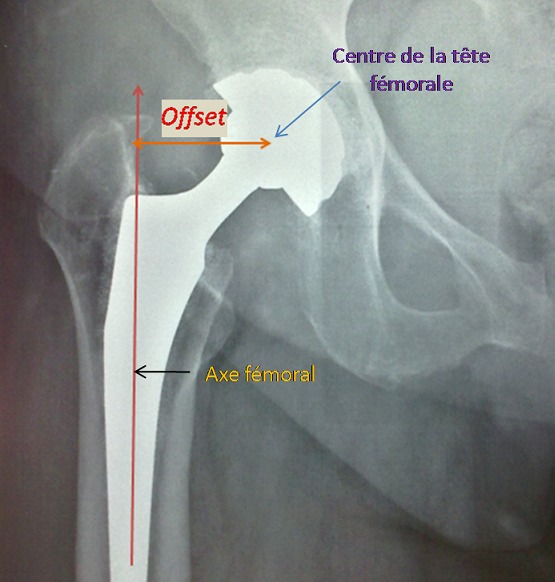
Mesure de l'offset fémoral

L’âge moyen était de 46 ans (18-75), et un sex ratio de 1,45 (16 hommes sur 11 femmes). L'indication de l'arthroplastie était surtout la coxarthrose primitive (Figure 1), avec 1 cas de luxation négligée de la hanche (Figure 2, Tableau 1). Toutes ces arthroplasties étaient implantées par voie postéro-externe de MOORE, la capsule était incisée le long du bord postérieur du moyen fessier et du grand trochanter, sans réinsertion des muscles pelvitrochantériens. Le diamètre de la tête fémorale prothétique en moyenne était de 50 mm (varie entre 42 mm et 58 mm), les tests de stabilité étaient validés par l'absence de luxation de la hanche en rotation interne de plus de 30°, à 90° de flexion (Figure 3). Sur les radiographies standards du bassin, l'offset fémoral postopératoire variait entre 36,2 mm et 52,3 mm, avec une moyenne de 44,25 mm (Figure 4, Tableau 2). La différence entre l'offset prothétique et l'offset fémoral controlatéral (sur une hanche saine) était de 5,1 mm (de -7,50 à +15,2). Il était augmenté dans 20 cas (91%) par rapport à l'offset controlatéral. Les résultats cliniques étaient évalués par le score de Merle d'Aubigné-Postel avec une moyenne de 17 points, et du score de WOMAC qu’était en moyenne de 15,2 points.

**Tableau 1:** Les indications de la prothèse totale de la hanche

Etiologies	Nombre des cas	Pourcentage
Coxarthrose primitive	18	66,6%
Ostéonécrose de la tête fémorale dans	5	33,3%
Fracture-luxation de la hanche négligée	1	3,7%
Coxarthrose secondaire sur une SPA	1	3,7%
Reprise prothèse de MOORE	2	7,4%

**Tableau 2:** Valeur de l'offset fémoral

	Offset du coté opéré	Offset du côté sain
Valeur moyenne pré-opératoire	38,5 mm	39,6 mm
Valeur moyenne post-opératoire	44,25 mm	39,6mm
Extrêmes	36,2 et 52,3 mm	36,75 et 59,45mm

**Figure 5:**
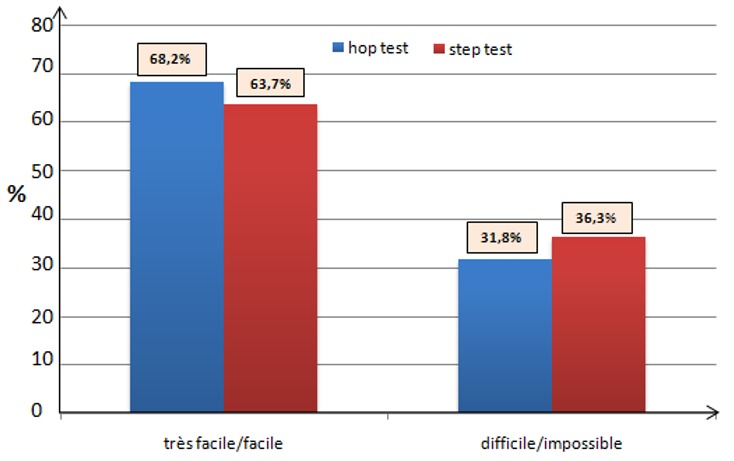
Résultats du hop et step test

Deux autres tests fonctionnels ont aussi été utilisés, à savoir le hop test (sauts répétitifs sur une jambe) et step test (monter sur une marche de 50 cm). L’évaluation était sur une échelle subjective afin de quantifier la faisabilité de l'exercice: très facile, facile, difficile, et impossible. Sept patients de notre série (31,8%) avaient une difficulté et/ou une impossibilité à effectuer le hop test, contre huit (36,3%) concernant le step test (Figure 5). L'analyse de la valeur de l'offset chez ce groupe de patient a objectivé une diminution significative par rapport au coté sain (Tableau 3). L'analyse de l'ensemble des résultats de tous nos malades a montré que les meilleurs résultats fonctionnels ont été obtenus chez les patients ayant eu une latéralisation de la tige fémoral avec un offset augmenté. Après un recul moyen de 19,5 mois (de 3 à 36 mois), Aucun cas de descellement n'a été objectivé à ce jour.

**Tableau 3:** Analyse comparative de l'offset en fonction du hop et step test

	Valeur moye de l'offset	Score de WOMAC	Score de Merle d'Aubigné-Postel
Groupe A	40,75 mm	24,2	13
Groupe B	47,75 mm	6,2	17

Groupe A : patients ayant une difficulté ou une impossibilité à faire le hop ou le step test

Groupe B : patients qui peuvent faire le hop et le step test

## Discussion

Il existe une très bonne corrélation entre l′offset et le bras de levier des muscles abducteurs de la hanche ainsi qu′avec la force de ceux-ci. Toute modification de l'offset influe sur l′angle d'attaque du moyen fessier et donc sur la force qui lui est nécessaire pour équilibrer le bassin. L'analyse de la littérature ne révèle pas assez de travaux scientifiques dans ce sens. Concernant la valeur approximative de l'offset fémoral, Massin et al. [4] ont observé une valeur moyenne d'offset fémoral de 41,0 ± 6,2 mm (de 20,5 à 59 mm) sur une série de 200 fémurs, alors que Noble et al. [5] ont identifié une valeur moyenne de 43 ± 6,8 mm (23,6 à 61 mm) sur 200 fémurs. Ces mesures peuvent être faites soit sur une radiographie standard de la hanche, ou bien sur des images de reconstruction d'une tomodensitométrie de la hanche. Rubin et al. [6] ont réalisé des mesures radiographique et tomodensitométrique sur 32 fémurs de cadavres et ils ont comparé celles ci à leurs mesures anatomiques, ils ont constaté que les radiographies standard (face et profile) donnaient des valeurs approximatives pour la caractérisation de la géométrie du fémur proximal, Cette équipe a trouvé une différence moyenne de 2,4 ± 1,4 mm entre les valeurs radiographiques et les valeurs anatomiques, alors que la TDM donnait une précision d'une valeur moyenne de 0,8 ± 0,7 mm, jugée meilleure que la précision radiographique. Cependant, la réalisation systématique d'une TDM pour une arthroplastie représente un surcoût peu acceptable en pratique, tandis que Debarge et al. [7], Unnanuntana et al. [8] et Suh et al. [9] considèrent que la planification 2D permet de prédire la taille du pivot fémoral et l'offset dans près de 70% des cas.

Dans notre série les mesures postopératoire de l'offset étaient sur des radiographies standards vu la difficulté de réaliser une TDM systématique pour tous nos malades, tout en prenant en considération l'agrandissement de l'image, la taille de la tète fémorale et en respectant la position des membres inférieurs en rotation interne au moment de la prise du cliché. La reproduction de l'offset fémoral reste un critère capital dans l'arthroplastie de la hanche; elle diminuerait le risque de luxation [10] et l'usure du polyéthylène [11], alors que son augmentation favorise la force des muscles abducteurs [12], améliore les amplitudes articulaires [1], diminue la boiterie et l'usage des cannes [13], Au prix d'un risque plus élevé de descellements liés à une augmentation des contraintes sur la tige selon Cannestra et al. [14] et Olofsson et al. [15], d'où l'intérêt d'une qualité du scellement irréprochable, ou bien préférer la fixation sans ciment qui semble moins sensible à l'augmentation des contraintes d'après Danesh-Clough et al. [16]. En revanche, sur le versant fémoral, la réduction de l'offset pourrait s'avérer bénéfique en favorisant les forces de compression axiale sur le composant fémoral. La latéralisation avec offset augmenté (à partir de 4 mm) est jugée comme la meilleure méthode par de nombreux auteurs pour retendre les parties molles en allongeant modérément le membre opéré [10].

## Conclusion

L'arthroplastie totale de la hanche reste toujours un sujet d'actualité et de débat, et les patients demandent de plus en plus une hanche fonctionnelle et anatomique. La restauration d'un offset normal est devenue une obligation dans le but d'avoir une hanche anatomique et fonctionnelle.

## References

[1] McGrory BJ, Morrey BF, Cahalan TD, Kai-Nan AN, Cabanela ME (1995). Effect of femoral offset on range of motion and abductor muscle strength after total hip arthroplasty. J Bone Joint Surg (Br).

[2] Lecerf G, Fessy MH, Philippot R (2009). Le déport fémoral (offset): concept anatomique, définitions, mesure, rôle dans la planification et la réalisation d'une arthroplastie de hanche. Revue de chirurgie orthopédique et traumatologique..

[3] Durand JC, Limozin R, Semay JM, Fessy MH (2003). Usure du polyéthylène à dix ans dans l'arthroplastie totale de hanche: Influence de l'offset fémoral. Rev Chir Orthop..

[4] Massin P, Geais L, Astoin E, Simondi M, Lavaste F (2000). The anatomic basis for the concept of lateralized femoral stem: A frontal plan radiographic study of the proximal femur. J Arthroplasty..

[5] Noble PC, Alexander JW, Lindhal LJ, Yew DT, Granberry WM, Tullos HS (1988). The anatomic basis of the femoral component design. Clin Orthop..

[6] Rubin PJ, Leyvraz PF, Aubaniac JM, Argenson JN, Esteve P, Deroguin B (1992). The morphology of the proximal femur a three-dimensional radiographic analysis. J Bone Joint Surg (Br).

[7] Debarge R, Lustig S, Neyret P, Ait Si Selmi T (2008). Confrontation de la planification radiographique préopératoire et des données postopératoires lors de la mise en place des prothèses totales de hanche non cimentées. Rev Chir Orthop..

[8] Unnanuntana A, Wagner D, Goodman SB (2009). The accuracy of preoperative templating in cementless total hip arthroplasty. J Arthoplasty..

[9] Suh KT, Cheon SJ, Kim DW (2004). Comparison of preoperative templating with postoperative assessment in cementless total hip arthroplasty. Acta Orthop Scand..

[10] Girard J, Vendittoli PA, Roy AG, Lavigne M (2008). Analyse de l'influence de l'offset femoral sur la function Clinique lors d'une étude prospective randomisée comparant les arthroplasties totales de hanche aux resurfacages. Ruvue de chirurgie orthopédique et réparatrice de l'appareil moteur..

[11] Ebied A, Hoad-Reddick DA, Raut V (2005). Medium-term results of the Charnley low-offset femoral stem. J Bone Joint Surg (Br).

[12] Yamaguchi T, Naito M, Asayama I, Ishiko T (2004). Total hip arthroplasty: the relationship between posterolateral reconstruction, abductor muscle strength, and femoral offset. J Orthop Surg..

[13] Bourne RB, Rorabeck CH (2002). Soft tissue balancing: The hip. J Arthroplasty..

[14] Cannestra VP, Berger RA, Quigley LR, Jacobs JJ, Rosenberg AG, Galante JO (2000). Hybrid total hip arthroplasty with a precoated offset stem: Four to nine-year results. J Bone Joint Surg (Am).

[15] Olofsson K, Digas G, Kärrholm J (2006). Influence of design variations on early migration of a cemented stem in THA. Clin Orthop Relat Res..

[16] Danesh-Clough T, Bourne RB, Rorabeck CH, McCalden R (2007). The mid-term results of a dual offset uncemented stem for total hip arthroplasty. J Arthroplasty..

